# MuSA: a graphical user interface for multi-OMICs data integration in radiogenomic studies

**DOI:** 10.1038/s41598-021-81200-z

**Published:** 2021-01-15

**Authors:** Mario Zanfardino, Rossana Castaldo, Katia Pane, Ornella Affinito, Marco Aiello, Marco Salvatore, Monica Franzese

**Affiliations:** IRCCS SDN, Via E. Gianturco, 113, 80143 Naples, Italy

**Keywords:** Data integration, Software, Computational biology and bioinformatics, Data mining

## Abstract

Analysis of large-scale omics data along with biomedical images has gaining a huge interest in predicting phenotypic conditions towards personalized medicine. Multiple layers of investigations such as genomics, transcriptomics and proteomics, have led to high dimensionality and heterogeneity of data. Multi-omics data integration can provide meaningful contribution to early diagnosis and an accurate estimate of prognosis and treatment in cancer. Some multi-layer data structures have been developed to integrate multi-omics biological information, but none of these has been developed and evaluated to include radiomic data. We proposed to use MultiAssayExperiment (MAE) as an integrated data structure to combine multi-omics data facilitating the exploration of heterogeneous data. We improved the usability of the MAE, developing a Multi-omics Statistical Approaches (MuSA) tool that uses a Shiny graphical user interface, able to simplify the management and the analysis of radiogenomic datasets. The capabilities of MuSA were shown using public breast cancer datasets from TCGA-TCIA databases. MuSA architecture is modular and can be divided in Pre-processing and Downstream analysis. The pre-processing section allows data filtering and normalization. The downstream analysis section contains modules for data science such as correlation, clustering (i.e., heatmap) and feature selection methods. The results are dynamically shown in MuSA. MuSA tool provides an easy-to-use way to create, manage and analyze radiogenomic data. The application is specifically designed to guide no-programmer researchers through different computational steps. Integration analysis is implemented in a modular structure, making MuSA an easily expansible open-source software.

## Introduction

Over the last decade, analysis of large-scale omics data has gaining a huge interest in predicting phenotypic conditions towards personalized medicine. Multi-omics data integration has become one of the main challenges in the era of precision medicine, particularly in oncology, due to high dimensionality and heterogeneity of data.

Consequently, powerful analytical methods and tools are required to handle multi-omics data throughout efficient strategies for data management, data linkage and data integration^1^. Therefore, the existing information systems need to be redesigned into advanced multi-layer data structures by integrating statistical and computational methods in order to optimize data management and analysis. Many methods have been developed to handle and analyze multi-omics data; however, they are mainly focused on data produced by molecular biology experiments overlooking the impact of other types of ‘omics, which also deserve attention. Huang et al.^2^ and Zanfardino et al.^1^ showed an in-depth review of multi-omics data integration methods and biological multi-omics integration tools. There is a significant number of tools that have been developed to handle multi-layers datasets^1,2^.

Some tools are specific for genomic data such as XENA UCSC browser^3^, Broad GDAC Firehose^4^, ModulOmics^5^ and mixOmics^6^. XENA UCSC browser is of particular interest because it allows visualization and analysis of both large public repositories and private datasets. Moreover, these tools have flexible architecture and support a variety of genomic data types providing a comprehensive view of genomic events within a cohort of tumors. Broad GDAC Firehose is only dedicated to public dataset, in particular to data managing from large scale projects such as TCGA. Indeed, Firehose projects make TCGA pre-processed data publicly available via web services and data portals. ModulOmics tool is able to identify de novo cancer driver pathways by integrating, into a single probabilistic model, multiple data types such as protein–protein interactions, mutations or copy number alterations, transcriptional co-regulator and RNA co-expression. Similarly, mixOmics offers several multivariate methods for the exploration and integration of biological datasets with a particular focus on variable selection. The tool mixOmics can use data from high throughput sequencing technologies (transcriptomics, metabolomics, proteomics, microbiome/metagenomics) but also data from some type of time-course or longitudinal omics data (such as spectral imaging), which are closer to our idea of integrating radiomic data in a classic multi-omics context.

One of the main limitations of these tools is the lack of integrating omics data with other data types, such as imaging and electronic health record data. In particular, medical imaging techniques, focused on the development of high-throughput algorithms to extract quantitative features from medical images, have evolved rapidly, leading to the development of radiomics^7^. Radiomics is a powerful tool in clinical research, especially when it is considered as additional source of features in a multi-omics biological environment. In this scenario, the increasing impact of non-invasive imaging techniques, in parallel with the evolution of bioinformatics tools, provides efficient methods for investigating the disease phenotype through the combination of radiomic features into a multi-omics biological framework.

In recent years, the relationship between the imaging features (imaging phenotype) of a particular disease and various genetic or molecular characteristics (genomic phenotype) has led to a new field of study defined “radiogenomics”, which combine imaging and genomics data^8^. Radiogenomics, in particular, aim to better identify non-invasive genotype associated to imaging phenotype, incorporating both phenotypic and genotypic metrics, in order to enhance precision in the early diagnosis and management of patients.

To date in the existing literature, there are few tools combining genomic and radiomic data, since integrating single-omics datasets from different domains in a concise manner can be a complex task. Gevaert et al.^9^ presented Imaging-AMARETTO tool to interrogate regulatory networks derived from multi-omics data within and across related patient studies for their relevance to radiography and histopathology imaging features predicting clinical outcomes. However, no handling data structure to better manage and organize multi-omics data, including radiomics, was considered in^9^.

Recently, data structures have also been designed for multi-omics assays. Examples of two recent data structures that handle multiple and heterogeneous data types from multi-assay genomic experiments are MultiAssayExperiment (MAE)^10^ and MultiDataSet^11^. In a recent study, Zanfardino et al.^1^ evaluated the data structure MAE to store and handle biological ‘omic and imaging data derived from different type of experiments, while maintaining the original representation of data and ensuring consistency between a single assay and clinical patient information during data sub-setting and analysis. In Zanfardino et al.^1^, an embryonal architecture of the presented tool was showed. However, in this study an easy, user-friendly graphical, modular Multi-omics Statistical Approaches (MuSA) interface is presented. Here, we describe its architecture and functionalities, then illustrating its capabilities using public breast cancer datasets from TCGA-TCIA databases as a radiogenomic case study.

## Results

Recently, we proposed to use MAE as a data structure to combine different omics data and experiments in order to facilitate the exploration of heterogeneous data derived from different domains. By using the MAE object, we set up operations such as: 1) selecting complete cases or sub-settings, 2) selecting samples with information in all datasets and/or in all ‘omics of a set of experiments, 3) selecting subjects with specific phenotypes and clinical outcomes. These operations can allow an easy management of multi-omics experiments and the correct alignment of assay/patients across different datasets, making radiogenomic integrative analysis more attainable. The use of MAE for these purposes requires users to have advanced knowledge of R, thus, limiting its valuable applicability in the medical and clinical fields. To overcome this obstacle and improve the usability of the MAE in the radiogenomic context, we developed MuSA tool that uses a Shiny graphical user interface (GUI) able to simplify the management and the analysis of radiogenomic datasets for a wider range of users.

### Application of MUSA on TCGA-TCIA Breast cancer data

**Figure 1:**
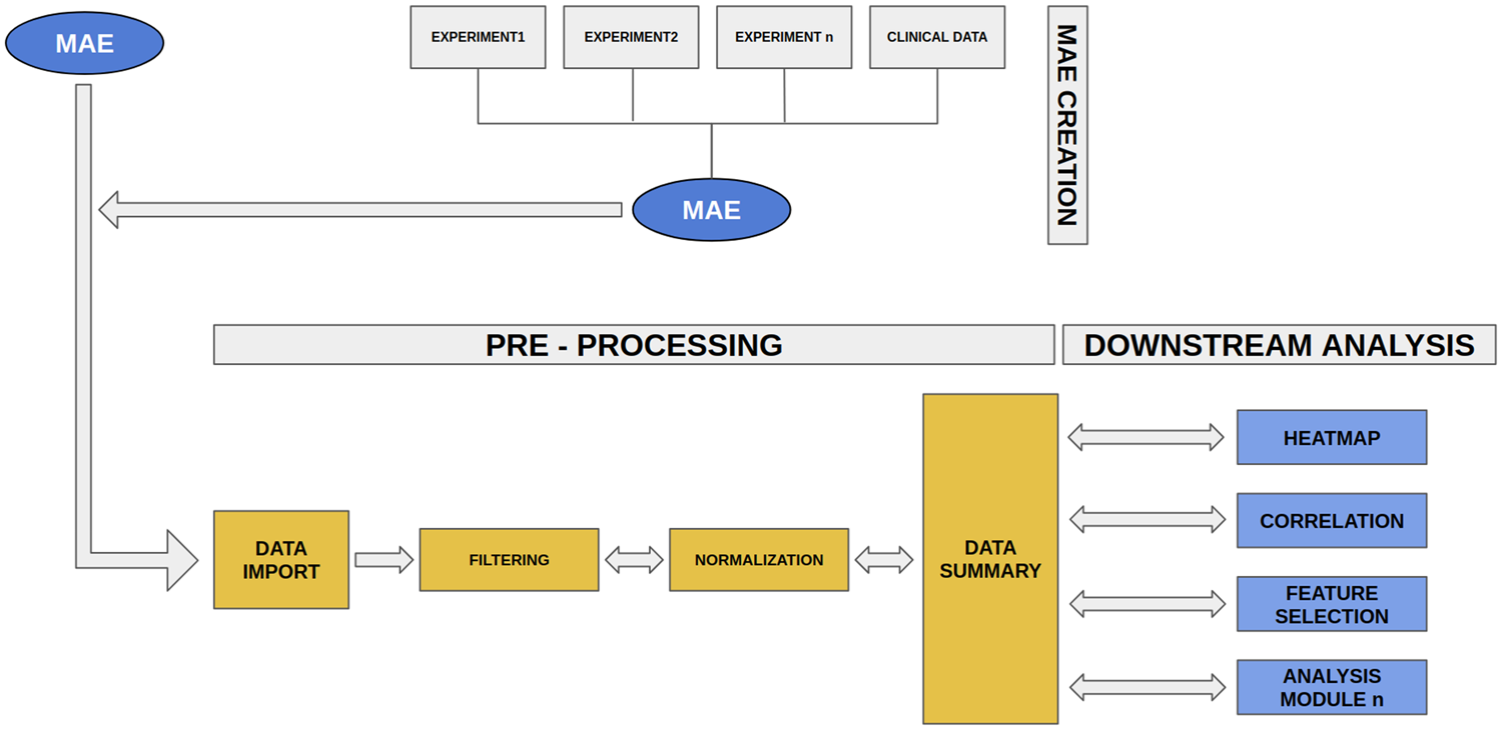
MuSA Architecture and Framework. Our architecture is modular and can be divided in two principal sections: pre-processing and Downstream analysis. In the first section, we also include the data upload module. The latter can accept both MAE or separated experiments (csv or summarizedExperiments) that will be transformed in a MAE by the MAE Creation sub-module. A MAE object is, thus, the input of Data Import module that provides a graphical summary of all experiments contained in the object. The next module in the workflow allows filtering the data by experiments, assays, features and complete data. Normalization module is optional, whereas Data Summary is mandatory. All modules that produce a tangible result are in the Downstream Analysis section. Double arrows between Filtering, Normalization, Data Summary and all Downstream Analysis modules indicate that the user can go back in the workflow and change the data environment (for example, deleting a feature or choosing another experiment). The results will change dynamically as in a classical Shiny application.

**Figure 2:**
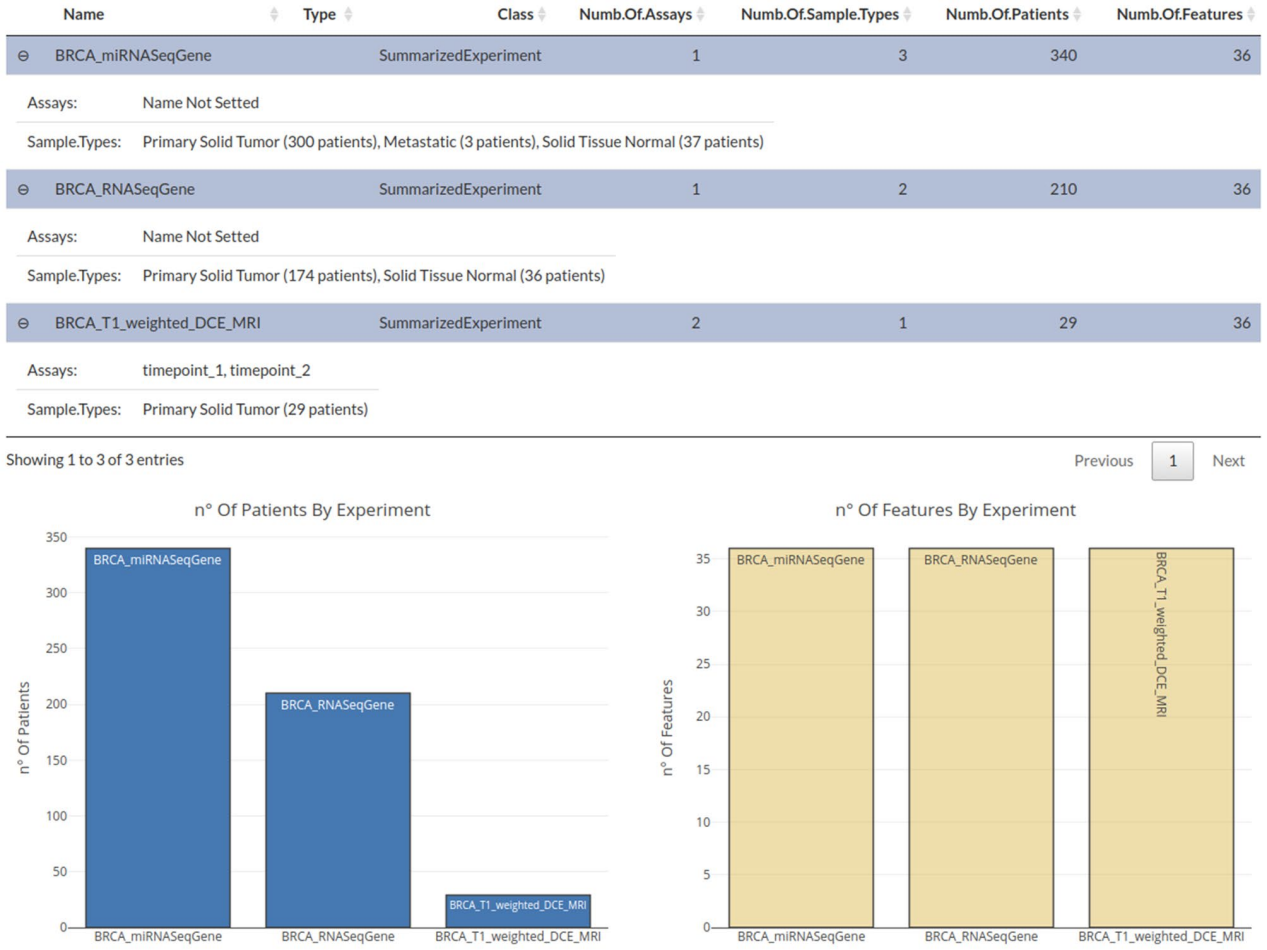
Data import summary. This Data Import tab shows the experiment data of the described radiogenomic case study. In the top table, the names of all MAE experiments are listed and for each of them, the assays (timepoint_1 and timepoint_2 in the case of BRCA_T1_weighted_DCE_MRI) and the sample types are reported. For each sample type, the number of patients is specified. The number of features and patients for each experiment are also represented as histogram (for a simple graphic representation, the number of features was limited to 36 for all experiments). The figure is generated by using R software version 3.6.1 (https://www.r-project.org).

**Figure 3:**
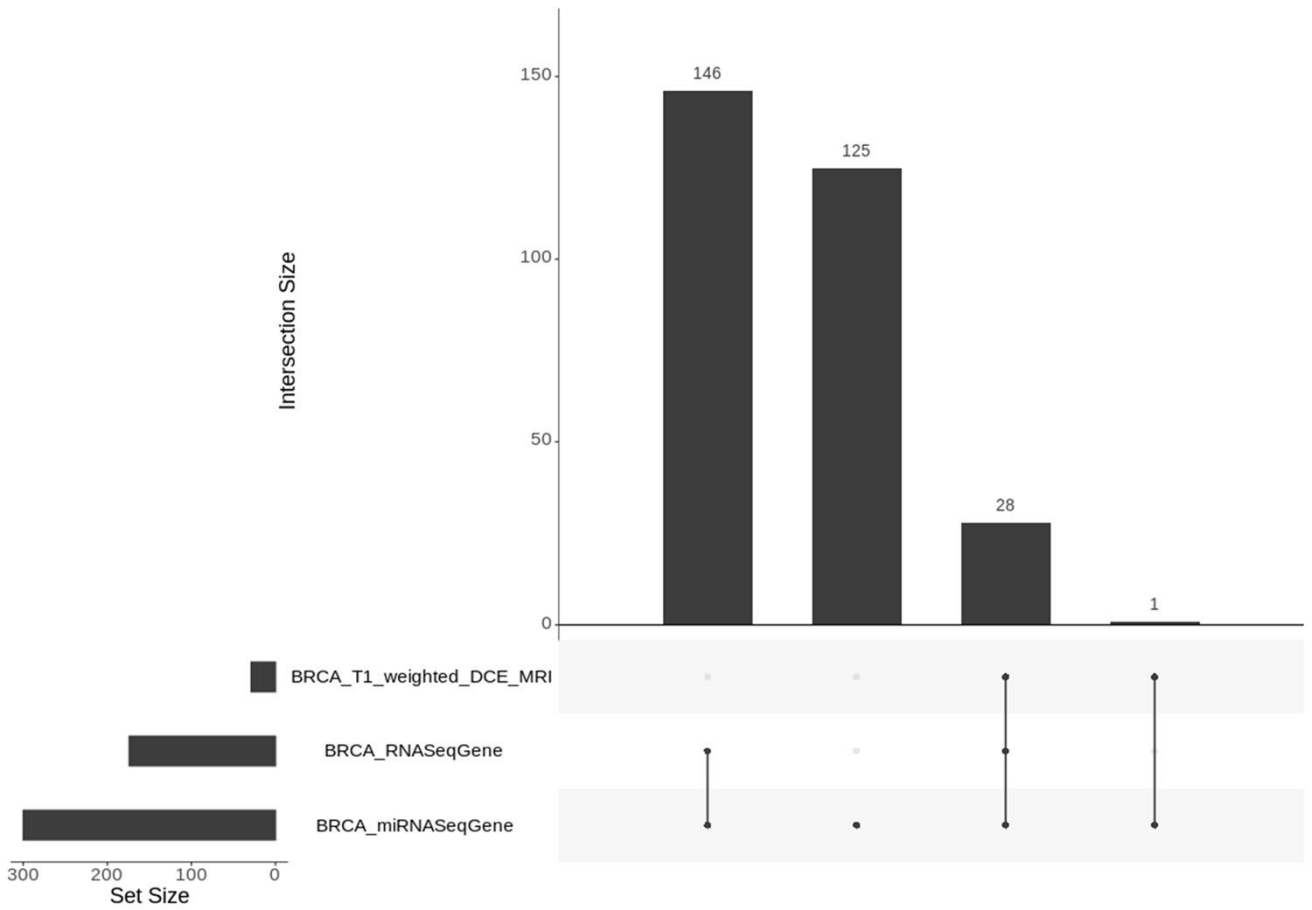
Generalized Venn diagram. A generalized Venn diagram for TCGA Breast Cancer sample membership in multiple assays performed by RNAseq, miRNAseq and Magnetic Resonance Imaging experiments. The visualization of set intersections was performed using the UpSet matrix design using UpSetR package. The figure is generated by using R software version 3.6.1 (https://www.r-project.org).

We illustrate the capability of MuSA in managing and analyzing multi-omics data using a publicly available TCIA dataset of a BRCA study^12^, which consist of 36 quantitative radiomic features extracted from primary tumor images of 91 patients, each acquired at a single time point in a tabular format and a TCGA dataset, BRCA study^12^, that consists of a MAE object (obtained through curatedTCGA R package^13^) composed by RNA-seq (expression quantification of 20,502 genes from 878 samples) and miRNA-seq (expression quantification of 1046 miRNA from 849 samples) experiments. We integrated TCIA and TCGA experiments together (using TCIA data transformed in a summarizedExperiment object) in a single MAE object following the workflow described in MAE vignettes on Bioconductor^14^. An appropriate module was developed to create a MAE starting from single experiments (csv or summarizedExperiment^15^); it was added in the home screen of MuSA to facilitate the development of the MAE data structure when the latter is not available. Several modules were developed to follow a precise data processing workflow, as reported in Fig. 1, that goes from the MAE upload to the downstream data analysis, based on back-forward process. A subset of our validation dataset is used to facilitate reproducibility on machines with low hardware capabilities and it is available on the GitLab page of MuSA (https://gitlab.com/Zanfardino/musa) to reproduce our case-study. The case study consists in a total of 36 features for each experiment and in 340, 210 and 29 patients for miRNA, RNA and imaging experiments, respectively. A screenshot of the “Data Import” module is shown in Fig. 2. In “Data Import” module, it is also possible to view a Venn diagram of patients with different experiments (Fig. 3).

**Figure 4:**
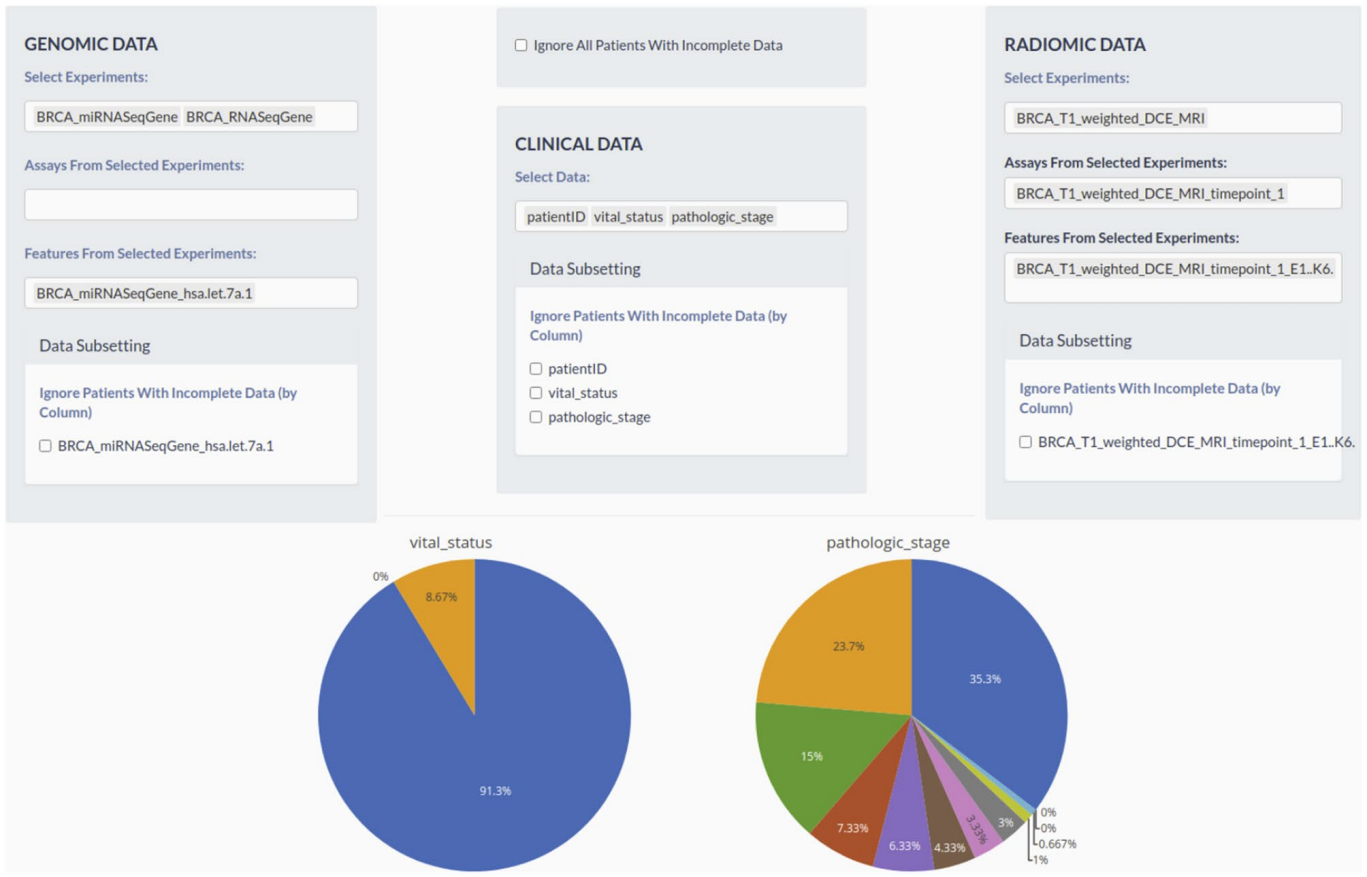
Filtering module. This module allows filtering the data by experiments, assays, features (clinical, radiomic and/or genomic) and complete data (overall – by “Ignore All Patient with Incomplete Data” or for a specific feature). Each type of data can be filtered separately through the specific sub-module. Each menu is dynamically built after a selection. For example, when the user selects an experiment, only features of the experiment are shown in the “feature menu”. A simple graphic statistic plot of clinical data is also shown in this module. The metadata plots show pre-filtering data per stage II. The figure is generated by using R software version 3.6.1 (https://www.r-project.org).

In the next module dedicated to data pre-processing and filtering, we selected clinical, genomic and radiomic features and applied the “Ignore All Patients with Incomplete Data” filter, which resulted in a selection of 19 patients with complete data (Fig. 4).

As exemplar, a specific clinical variable, breast tumor stage II, was selected for the 19 patients. In particular, stage II breast cancer is considered invasive, meaning that the breast cancer is growing, but it is still contained in the breast or growth has only extended to the nearby lymph nodes. This stage is divided into groups: stage IIA and stage IIB. The difference is determined by the size of the tumor and whether the breast cancer has spread to the lymph nodes^16^.Therefore, MuSA tool, in this specific case study, was used to explore possible features association of radiomic and genomic cases in stage IIA (35.3%, Fig. 4) and stage IIB (23.7%, Fig. 4).

**Figure 5:**
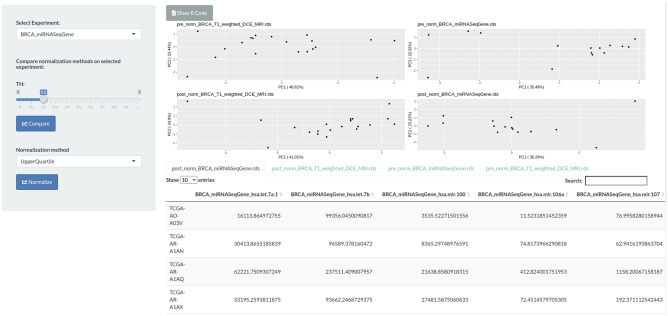
Normalization module. In the Normalization module, the user can choose the normalization method to apply on each single experiment. A PCA plot is displayed for each pre- and post-normalization experiment. Moreover, the table with all processed data is displayed at bottom of the main page. This step is optional. In this example, radiomic and genomic data are normalized via upper-quartile normalization method per tumor stage II. The figure is generated by using R software version 3.6.1 (https://www.r-project.org).

**Figure 6:**
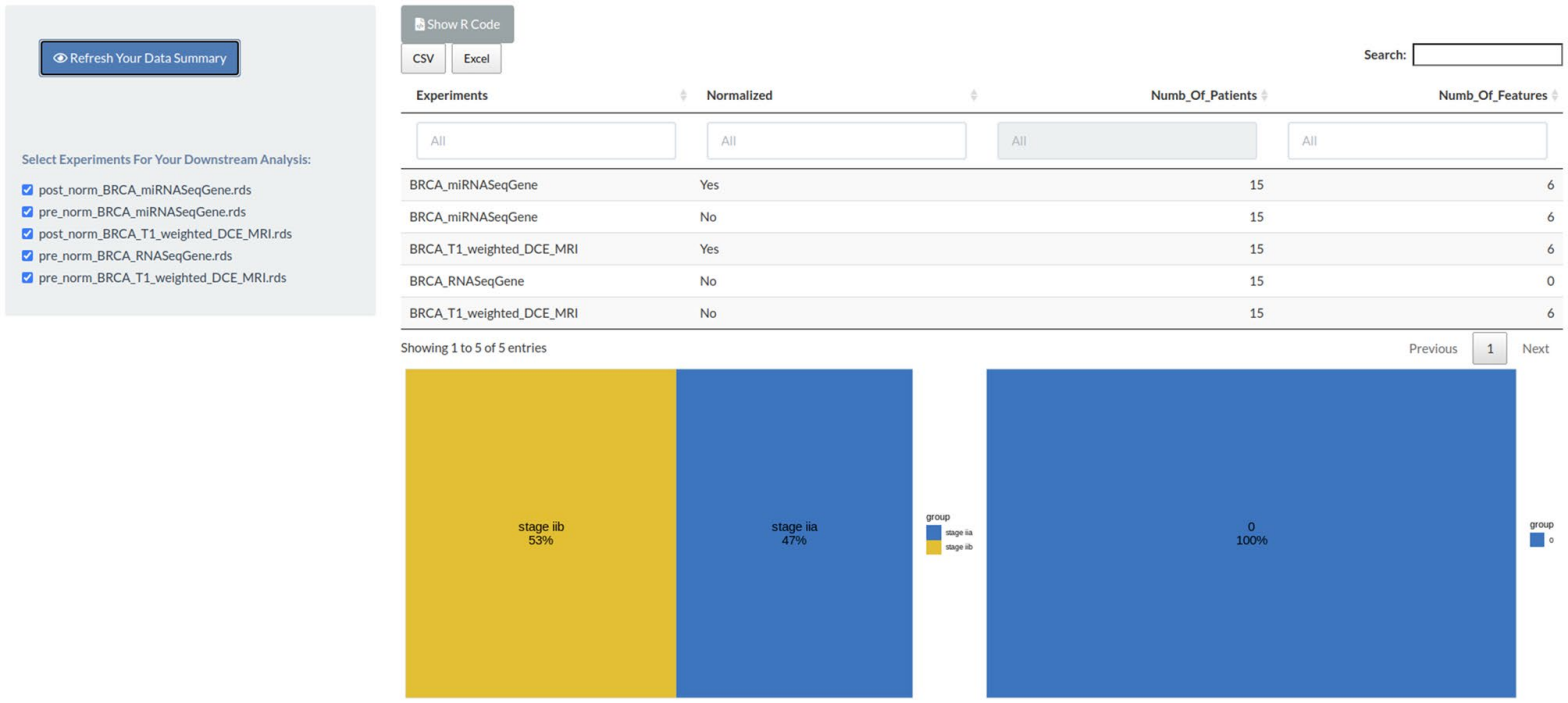
Data summary module. In the Summary module, a review of all available experiments is shown after the operation executed in the workflow. In this module, the user may decide to execute a downstream analysis in the next step or go back to change data selection or normalization methods. If the user decides to use a downstream analysis module, using the left checkbox list, the final experiments need to be selected as input for the next module. In this section a graphic statistic plot of clinical data is also reported. In this example, the percentage of data available after filtering is reported for tumor stage II. The figure is generated by using R software version 3.6.1 (https://www.r-project.org).

All data associated to the selected experiments could be normalized through the “Normalization” module (Fig. 5) and passed to a summary view that allows to summarize the available experiments, features and clinical data, which are ready to be analyzed in the next modules (Fig. 6). For the specific case study, both radiomic and genomic features were normalized using the upper-quartile normalization method.

**Figure 7:**
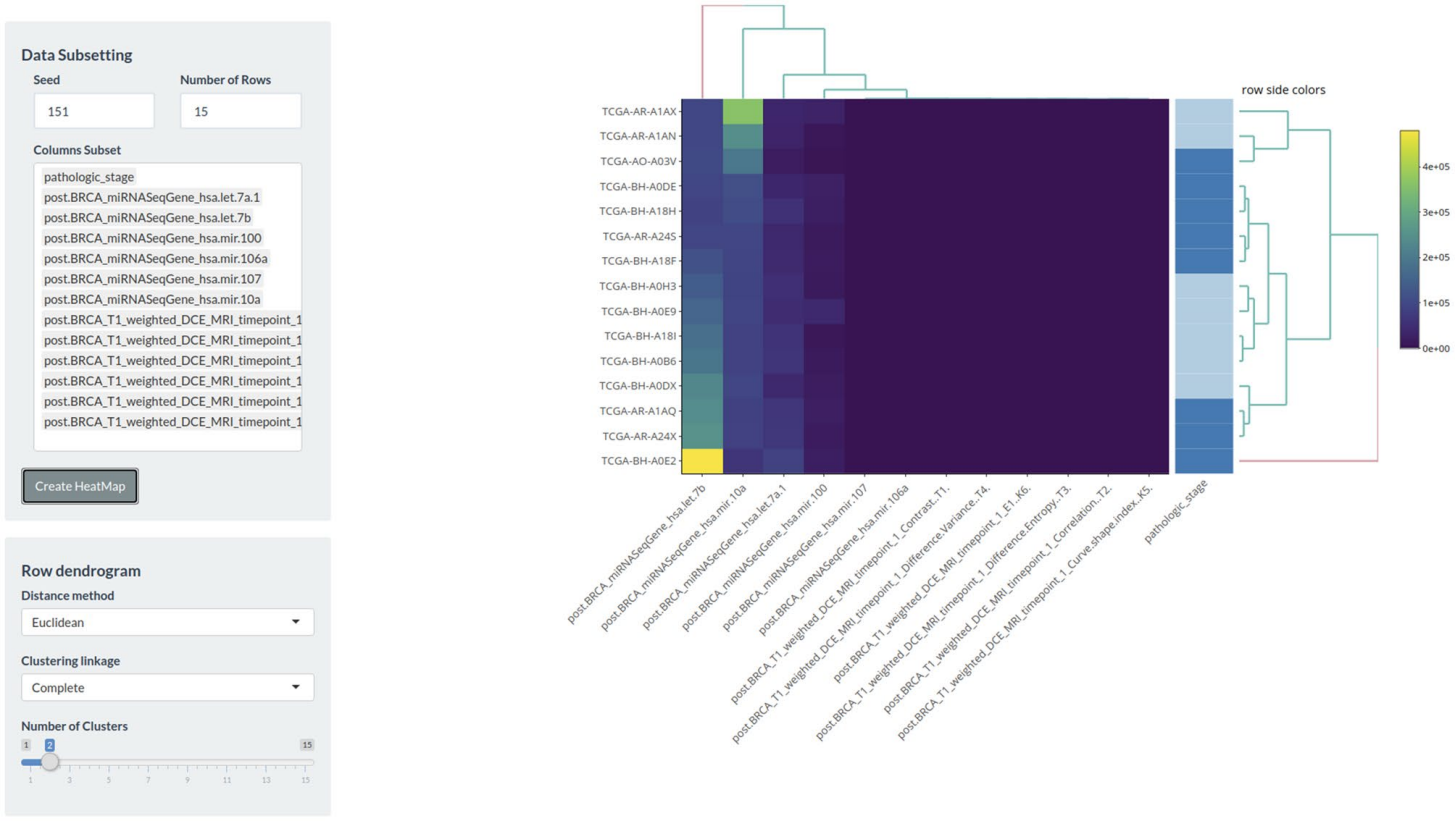
An example of downstream analysis module. Heatmap module is one of the downstream analysis modules. In this case, on the left-hand side, the user can choose the features to view in the heatmap and can set distance method, i.e., clustering linkage, number of clusters and other parameters that also define the graphical aspect of the heatmap. Other downstream analysis modules are based on the same structure. The figure is generated by using R software version 3.6.1 (https://www.r-project.org).

**Figure 8:**
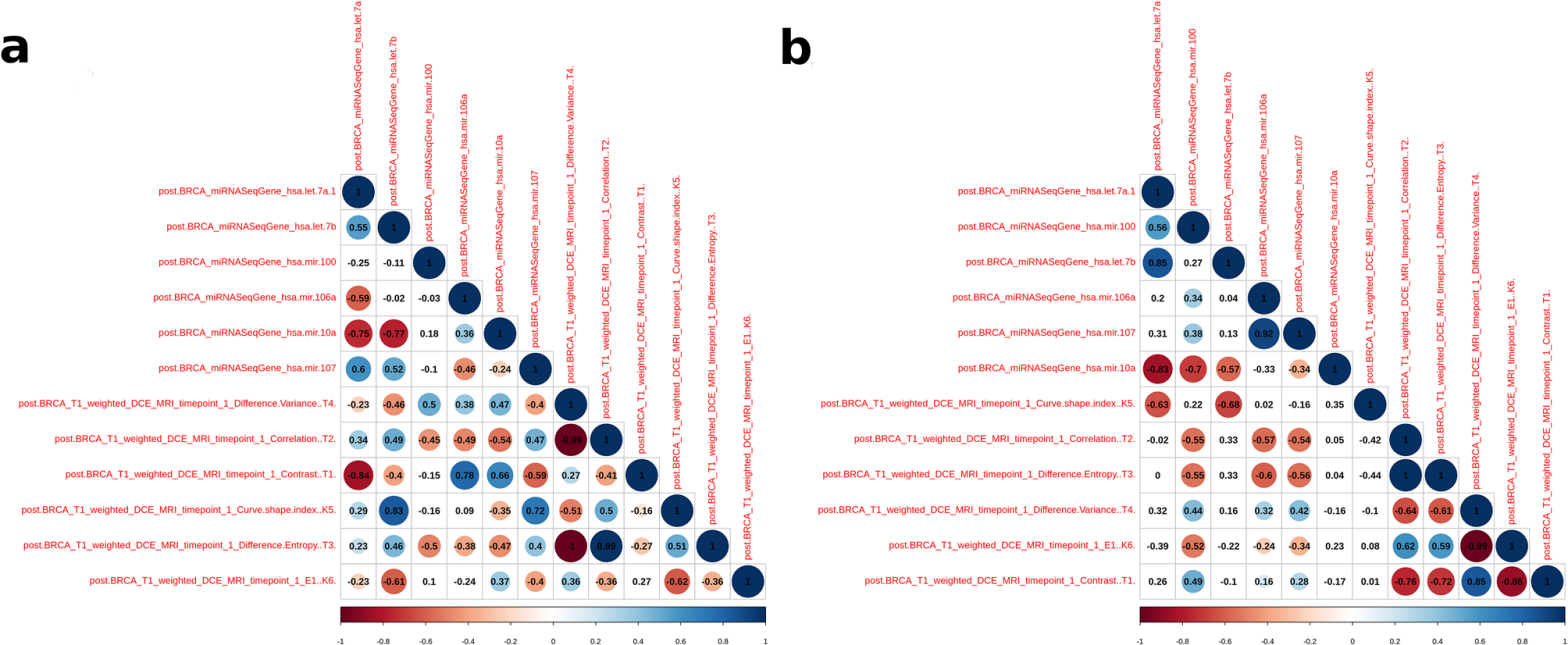
An example of correlation analysis for stage II. Correlation module is one of the downstream analysis modules. In this case, on the left-hand side (**a**), we extracted the correlation plot between radiomic and genomic features for stage IIA. In stage IIA, T1_weighted_DEC_MRI contrast shows a significant (*p* value < 0.05) negative correlation (rho = -0.84) with miRNA_hsa.let.7a; whereas T1_weighted_DEC_MRI_curve_shape_index is significantly (*p* value < 0.05) correlated (rho =  + 0.83) with miRNA_hsa.let.7b. On the right-hand side (**b**), we extracted the correlation plot between radiomic and genomic features for stage IIB. In stage IIB, T1_weighted_DEC_MRI_curve_shape_index shows significant (*p* value < 0.05) positive correlations with both miRNA_hsa.let.7a and miRNA_hsa.let.7b. The figure is generated by using R software version 3.6.1 (https://www.r-project.org).

Downstream analyses of all selected multi-omics features were performed through “Heatmap” (Fig. 7) and “Correlation Plot” (Fig. 8) modules (from “Downstream Analyses” section), respectively in order to quickly and graphically highlight potential association between all selected features of interest.

Through MuSA tool, we extracted the correlation plots between radiomic and genomic variables in stage IIA and IIB, as shown in Fig. 8. By simultaneous exploring genomic and radiomic features for tumor stage, we can observe that a number of features are significantly correlated in both stage IIA and IIB. Breast radiogenomics can, therefore, initially demonstrate how imaging parameters, when assessed with genomic features, can significantly contribute to our understanding of tumor biology and highlight the potential for radiogenomics in breast cancer.

**Figure 9:**
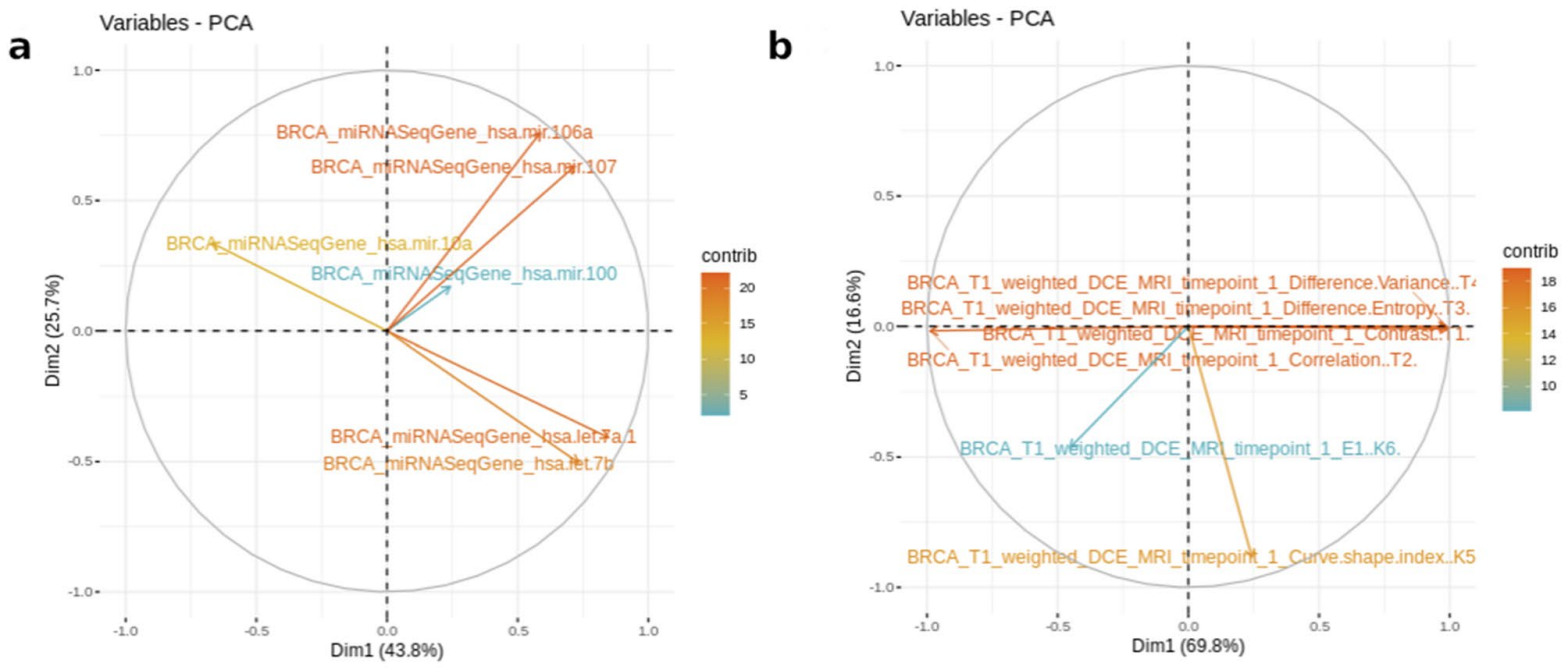
An example of feature selection module via PCA for stage II. Feature Selection module is one of the downstream analysis modules. In this case, on the left-hand side (**a**), we extracted the PCA correlation circle plot for radiomic in stage II. On the right-hand side (**b**), we extracted the PCA correlation circle plot for genomic features in stage II. The most important or contributing variables are highlighted on the correlation circle plot for PCA. The figure is generated by using R software version 3.6.1 (https://www.r-project.org).

The “Feature Selection” module can also be used to prepare data for a downstream analysis, for example, the application of machine learning (ML) techniques. Figure 9 shows the output of MuSA tool by applying PCA method in both radiomic and genomic features for stage II, in order to initially reduce the number of variables or observe the explained variance and contribution of the features to two dimensions.

Furthermore, users can freely choose the modules to use according to their need. For example, it is possible to skip data normalization if the dataset is already normalized. For more details on all options see Materials and Methods section.

#### Execution times

MuSA is CPU-based and execute each step with the following time:

- Uploading MAE and showing summary table and graphs: 1.86 s.
- Ignoring all NA patients: 3.12 s.
- Adding a feature to selection: 1.4 s.
- Normalizing (e.g., Upper-quartile) and showing summary table and plots of results: 4.36 s.
- Generating the Heatmap: 2.13 s.
- Running Correlation and generating the plot: 0.83 s.
- Running Feature Selection and generating the plots: 0.75 s.

These execution times were obtained from a mean of 10 executions for each step on a machine with Ubuntu 19.10 (64-bit), CPU 4.20 GHz × 8, 64 GB RAM, using a MAE dataset of three experiments with a total 579 patients and 36 features for each experiment. The normalization, heatmap and correlation steps were executed on a filtered dataset of 19 patients/11 features.

## Discussion

The availability of heterogeneous datasets from radiomics and genomics has highlighted the need to evaluate specific models and methods for storing, managing and analyzing multi-omics data in order to better understand disease mechanisms and to explore the relationship between multiple molecular and phenotypic layers. Despite several data structures based on multi-layer architecture have been proposed, so far, none of these has been designed and assessed to include radiomic data as well. To address this need, we recently proposed to use MAE as a suitable structure to manage imaging and genomics experiment data. MuSA will be useful to the community of biomedical and bioinformatic researchers for applications to oncological studies and other complex diseases with available multi-omics, radiomic features, and clinical data. In particular, in the context of the omics community, the main use of our tool is to manage and organize a radiogenomic experiment as well as investigate multiple heterogeneous layers by executing preliminary explorative analysis, specially to consider feasibility and applicability of a design experiment and to build an integrated dataset that can be ready to run in a complex analysis pipeline.

In our previous study^1^, we demonstrated that is possible to handle imaging genomics experiment together, in a meaningful manner, although they were generated by distinct analytic fields. Here, we developed a user-friendly tool to explore radiogenomics data in a visual framework. We developed a flexible GUI-based Shiny application, referred as MUSA to support physicians in designing radiogenomic studies. This application is specifically designed to guide no-programmer researchers through different computational steps available in the described data processing workflow, which also includes downstream analysis modules. Moreover, integration analysis was implemented in a modular structure and based on back-forward process. Therefore, other available approaches in the literature could be easily encapsulated in further releases.

Already existing tools use multi-omics data for specific scopes such as using regulatory networks to predict clinical outcomes (Imaging-AMARETTO^9^), variable selection (mixOmics^6^), provide pre-processed data from public databases (Broad GDAC Firehose^4^) or allows visualization and analysis for public and private dataset (Xena UCSC browser^3^). MuSA is set in this context as a tool that is able to cover some of these scopes but especially to be data independent (not only TCGA or public data) and data type independent (not just classic omics such as genomics, transcriptomics, etc., but also radiomic data). Moreover, the flexibility and expandability features allow the user to adapt MuSA tool for specific analysis. In fact, compared to other imaging genomics tools, such as Imaging-AMARETTO^9^, MuSA allows users to add any customized methods, to better manage multi-omics data structure, including radiomic features, through the creation of a MAE structure, to pre-process and normalize data, which are key steps to reduce bias and systematic errors in the downstream analysis.

Indeed, in this work we filled a gap in the existing literature by providing a user-friendly tool which is able to combine different omics data and experiments in order to facilitate the simultaneous exploration of heterogeneous data derived from different domains. Therefore, at this stage, we added exploratory statistical methods to facilitate initial data discovery across multi-omics data.

In the next release, implementation of specific statistical methods used in the analysis of radiogenomic data will be added. In a new version of the tool, we will present other feature selection methods such as filter based method, embedded based method and combine methods along with several ML methods. This will allow to systematically evaluate a set of suitable feature selection methods and learning algorithms, which are critical steps to develop clinically relevant models, since there is no “one fits all” approach, but performance of various feature selection methods and learning algorithms have been shown to depend on application and/or type of data^17–21^. Therefore, we intend to develop a full-integrated module that executes a complete machine learning analysis inside the application, giving the possibility to split the dataset resulted from the current version of MuSA in test-train sets, to choose from a list of different type of classifier and regressor and to reports graphical summary of results and metrics. Several ML algorithms will be implemented through training, validation (internal validation, e.g., cross-validation) and testing (external validation) procedures, possibly bootstrapping. Model performance will be measured in terms of the receiver operating characteristic (ROC) curve, or the area under the ROC curve (AUC) and Brier score, the mean squared prediction error.

On the other hand, although MuSA already implements several methods to facilitate multi-omics data management and analysis, more work still needs to be done to speed-up the time-demanding computations required for carrying out some specific steps, such as filtering and visualization of a large amount of data. A possible improvement could be the split-up of the computations on multiple cores/CPUs. A faster MuSA may also implement modules based on existing tools such as TCGAbiolinks^22^ and curatedTCGA^13^ that would allow to directly download and prepare the data. An additional value in the next release will be also provided by adding a GUI for automatic data downloading from databases such as TCGA.

To promote the reproducibility of research, an important feature of MuSA, linked to computational reproducibility, is the possibility to display the code that generate the results, which is very challenging for tools based on GUI^23^. For this reason, MuSA guides the user to organize and analyze heterogeneous data, automatically stores input/output data and allow to copy the executed code behind the followed workflow.

In the next versions of MuSA, we also envisage the development of functionalities to automatically produce a comprehensive analysis report that includes final results and intermediate data. The major capabilities of MuSA are illustrated by analyzing several publicly available TCGA-TCIA datasets merged in a single MAE object. In this context, we have not considered mutation datasets that are generally represented as RaggedExperiment^24^, a flexible representation of copy number, mutation and other data that fit into the ragged array schema for genomic location data. The possibility of including this type of data requires the management of new layer of information based on genome coordinates. The management of this functionality through the graphical interface will be released in the next version.

Moreover, we will add an extra value to the tool by integrating through Shiny library the possibility to import and process imaging data. In particular, imagining preprocessing, ROI or VOI segmentation, interpolation, resegmentation, image discretization, and finally feature calculation and calibration steps will be performed accordingly to the existing guidelines^25,26^.

## Materials and methods

### MuSA architecture and framework

#### Graphical user interface

The graphical interface has been designed to guide the user during the building of a “ready to analyze” radiogenomic data and to run preliminary analysis on the datasets. To this purpose, the upper part of the interface displays the navigation bar illustrating all modules, each of them representing a management/analysis step in a consecutive order (not all steps are mandatory). Each module contains more specific functions (left-hand side of the interface) acting on the dataset in a dynamic mode. The results of the functions (tables and plots) are reactively shown in the main panel, which is displayed on the right-hand side of the interface. All the tables (.csv or .xlsx) and plots (.png) can be downloaded with specific buttons by the user. Moreover, tables and some plots are interactive and allow exploring the data more deeply. All the structures of the GUI were developed with Shiny (v1.5)^27^, Shinyjs (v1.1)^28^ and Shinydashboard (v0.7.1)^29^ R packages. The tables and the plots were built by DT v0.14 (a wrapper of javascript library ‘DataTables’)^30^ and plotly (v4.9.2.1) / ggplot2 (v3.3.2)^31,32^, respectively.

#### Getting started for the analysis

The only step required before the execution of MuSA is the set-up of a configuration file in which the experiments must be tagged as “Genomic” or “Radiomic”. This step is mandatory for the correct management of all data and features throughout the MuSA workflow. Also, when the MAE is created via the interface, each experiment must be tagged in the config file and in this case the name of experiment will be the file name. The application is designed to be run both as a single-user or server-side application. When MuSA is executed, a new temporary project folder will be created, as described in “Data format and Data organization” section and all tables, plots and results are stored in this folder.

#### Data format and data organization

MuSA is based on the usage of MultiAsssayExperiment data format^1,10^ to store multi-omics experiments and data. The inputs of MuSA can also be comma separated values or summarizedExperiments^15^. Therefore, any genomic data, unless genome variants, and extracted radiomic features can be uploaded in MUSA tool as both MAE or separated experiments (tabular format, i.e., csv or summarizedExperiments). Indeed, both formats of data can be used to create a new MAE that can be passed to the designed modules in the workflow of the interface. For each selected or created experiment (e.g., it can also be a derived experiment such as the one created by normalization step), MuSA creates a list of available experiments based on data saved in a temporary and session-specific directory. This allows the user to decide which data to select for post-processing and downstream analysis. The objects of plots are also saved in this folder and, together with the objects of all created experiments, they can be opened in a R/Rstudio session and used by R advanced users for further analysis (all data are saved as R Data Format—rds).

#### Modularity and reproducibility

MuSA is implemented using Shiny library in a modular structure. Indeed, the Shiny package easily allows developing advanced and practical interfaces in a web-based approach thanks to the split of code in the Shiny User Interface that provides all the aesthetic components that the user interacts with and the Shiny Server Side performing the required computations. Moreover, it is possible to implement complex applications by combining multiple independent modules. In our case, each “Integration Analysis tab” interface corresponds to a different module that runs as a black box with a common input and a specific output. This modular structure helps handling the complexity of the interface and better facing the maintainability of the software. This is relevant when novel analysis modules need to be added. In fact, in order to add a novel module, it will only be necessary to incorporate a novel tab which implements the required functionalities and presents a compatible input interface. Thanks to this choice, MuSA results in an easily expansible software. This will allow users to add any customized methods for different modalities (experiments). MuSA is implemented with functionalities that allow code reproducibility through the possibility to display code for each step of the analysis. For this functionality we used Shinymeta (v0.2.0) (https://github.com/rstudio/shinymeta). Moreover, a Docker container, that works on different hardware architectures and operating systems, is available.

### Analysis workflow

#### Pre-processing and filtering

The pre-processing step consists of a series of required operations for the proper execution of downstream analysis steps and to build a dataset ready to be analyzed, even in an external pipeline. Such operations allow MuSA to access the information stored in the MAE structure and to prepare the required environment. Many operations in this step occur in the background and without the user intervention. One of these is the creation of a session-linked temporary folder used to store intermediate results of the MuSA workflow. The first user operation is the building or the uploading of MAE object. The building is based on the workflow described in MAE vignettes on Bioconductor^8^. At the end of the pre-processing, MuSA produces a summary of all available data in the different MAE experiments and allows the user to select and filter the data. In the summary tab, for each experiment, the available sample types are also shown as one of the types defined by The Cancer Genome Atlas Program (https://gdc.cancer.gov/resources-tcga-users/tcga-code-tables/sample-type-codes). The filtering step helps choosing the experiments, assays and features through specific boxes dedicated to clinical, genomic and radiomic data. A separate module for manual feature selection was added to the tool to facilitate the filtering of row features when a large number of features are presented in the experiments (e.g., 20,502 RNA-seq features). The module takes as input a comma separated list of features that are then automatically selected in the GUI. Moreover, the user can also “Select all” features with just one click. The filtering step also allow to remove eventually NaN data using specific checkbox that allows the user to drop rows (patients) or columns (features) with incomplete data. An option to remove all incomplete data (all patient with at least one incomplete feature) is also available. However, imputation is a more preferable option rather than dropping because it preserves the data size. Therefore, the user also can select a threshold for imputation and decide to fill the missing values of a certain features with the mean value of that feature. During the filtering step, a dynamic table shows the available patients for the actual setting and a plot tab displays statistics on the associated clinical data.

#### Statistical approaches

Five normalization techniques were implemented in MuSA tool and they can be applied to radiomic and genomic features. Data normalization methods are important in both radiomic and genomic field, due to their inner differences in range, scale and statistical distributions. Non-normalized features might present high levels of skewness, resulting in misleading low *p* values in statistical investigation^33,34^. Lately, normalization techniques are of great interest in data preprocessing especially for machine learning algorithms^35^. In fact, overlooking feature normalization step may lead to individual features being over or underrepresented and eventually introduce bias into developed models and statistical analysis^34^.

In MuSA tool, radiomic and genomic features can be normalized accordingly to five most common normalization methods. Min–Max normalization, Z-score normalization, log2 normalization, upper quartile and whitening methods were included in this step. Min–max normalization is one of the most common method to normalize data. For every feature, the minimum value of a feature gets transformed into a 0, the maximum value gets transformed into a 1, and every other value is rescaled to lie within a range of 0 to 1^36^.Regarding standardized z-score normalization, each feature was normalized as z = (x-$\stackrel{-}{x}$)/s, where x, $\stackrel{-}{x}$ and s are the feature, the meanand the standard deviation respectively^37^. Features can also be standardized as Log-transformation (base 2), a constant value *a* = *b – min(x)* where *b* is 1 and *x* is the feature, was added to the data for handling negative values^38^. The upper quartile normalization divides each read count by the 75th percentile of the read counts in its sample^39^; lastly, whitening normalization technique from the principle component analysis (PCA), is based on a linear transformation that converts a vector of random variables with a known covariance matrix into a set of new variables whose covariance is the identity matrix, meaning that they are uncorrelated and each have variance equal to one^40^.

Thanks to the modularity structure of MuSA tool, the user can add any customized normalization method.

Moreover, MuSA also provide a comparison function among the normalization methods to help the users deciding which method is more suitable for their need. The function takes as input the normalized datasets and, by applying a Principal Component Analysis, it investigates the cumulative variance to select the minimum number of Principal Components (PCs) for each normalization method. Cumulative variance threshold can be chosen by the user. The default value is usually 80%. The normalization method that uses few PCs to express the desired cumulative variance could be chosen as the best method.

#### Downstream analysis and integration

MuSA downstream analysis supports the user in the visualization (graphical and tabular) of all available experiments after the pre-processing and the normalization steps. Indeed, the latter creates normalized copies of the initial experiments that can or cannot be used by the user. Therefore, before using a downstream analysis module, an overview on the type, number and characteristics of all data is displayed in the mandatory “Data Summary” module. Here, the user can also choose, through a checkbox, the experiments to be used as input of the downstream analysis modules. This operation allows to have a standard input for all the modules dedicated to the data analysis, making our tool modular and scalable. In our first version of MuSA, we made available three different downstream analysis modules dedicated to data integration and exploration: clustering analysis (i.e., heatmap), correlation analysis and feature selection. False discovery rate (FDR) was taken in consideration in the explorative analysis included in MuSA. False discovery rate (FDR) correction was applied, and q-values are reported. The Heatmap modules is based on heatmaply v1.1.0^41^, an interactive cluster heat maps using plotly^31^. Correlation analysis is based on corrplot v0.84^42^, while feature selection module, which includes SVD (Singular Value Decomposition) and PCA (Principal Component Analysis), is based on svd v0.5 (The R Base Package), factorMineR v2.3^43^ and factoextra v1.0.7^44^. Feature selection module performs unsupervised feature selection by using PCA or SVD. These methods can be applied to the single experiments, depending on the user’s need, and are often useful to reduce the number of variables^1,45^.For each of these modules, different user’s options are available to dynamically show different results. In the heatmap module, for example, distance methods, clustering linkage, number of clusters and other inputs, including graphical settings, are available.

### Validation Dataset (TGCA-TCIA)

A publicly available dataset was used as validation data of MuSA. Among sources of multi-omics data, The Cancer Genome Atlas (TCGA, https://tcga-data.nci.nih.gov/tcga/ ), along with The Cancer Imaging Archive (TCIA, https://www.cancerimagingarchive.net/access-data/)^46^, is the largest data repository in cancer research. In fact, TCGA-TCIA data have become fundamental for radiogenomics cancer studies^47^.

We used The Cancer Genome Atlas Breast Invasive Carcinoma (TCGA-BRCA) data collection. The TCGA-BRCA project is focused on linking cancer phenotypes to genotypes by providing clinical images corresponding to subjects from TCGA^12^. This allows researchers to explore the TCGA/TCIA databases for association among tissue genotype, radiological phenotype with patient outcomes^48^.

We exploited clinical data, radiomic data, genomic measurement including mRNA gene expression and microRNA for breast invasive carcinoma. The download of genomic measurements has been performed using TCGA Assembler tool^49^, free software R (R version 3.5.2)^50^ and TCGAbiolinks R package.

TCGA breast invasive carcinoma RNA sequencing (RNAseq) uses a platform based on the Illumina system. The TCGA deposited data contains information about both nucleotide sequence and gene expression. RNA sequence alignment provides different levels of information such as RNA sequence coverage, sequence variants (e.g., fusion genes), expression of genes, exon, or junction. The RNAseq dataset counted expression quantification of 20,502 genes from 878 samples.

TCGA breast invasive carcinoma miRNA expression quantification data is also produced on Illumina HiSeq 2000 sequencers (Illumina Ventures, San Diego, CA, USA). TCGA-formatted miRNA-seq data according to the BCGSC miRNA Profiling Pipeline produces expression quantification of 1046 miRNA from 849 samples.

TCIA breast invasive carcinoma imaging yields a total of 91 cases with Magnetic Resonance (MRI) images. In order to minimize variations in image quality across the multi-institutional cases we included only breast MRI studies acquired on GE 1.5 T magnet strength scanners (GE Medical Systems, Milwaukee, Wisconsin, USA) scanners. The dataset of biopsy proven invasive breast cancers included 74 (88%) ductal, 8 (10%) lobular and 2 (2%) mixed. Of these, 73 (87%) were ER+, 67 (80%) were PR+ and 19 (23%) were HER2+^51^.

The complete dataset of the 36 MRI extracted features was downloaded from the TCGA Breast Phenotype Research Group Data sets (available at: https://wiki.cancerimagingarchive.net/display/DOI/TCGA+Breast+Phenotype+Research+Group+Data+sets, accessed October 9, 2019).

## Data Availability

Project name: MuSA; Project home page: MuSA is freely available on GitLab at https://gitlab.com/Zanfardino/musa;web-based version of MuSA is also available at http://188.166.63.238:3838/MuSA_0.6/ . Programming language: R and JavaScript; Other requirements: Shiny 1.5 or higher*.*
